# Supplementary material for: Preventability of unplanned readmissions within 30 days of discharge. A cross-sectional, single-center study
Source: PLoS One. 2020 Apr 2;15(4):e0229940. doi: 10.1371/journal.pone.0229940 (PMC7117704; doi:10.1371/journal.pone.0229940)
Supplement: S1 Table — (DOCX) [file pone.0229940.s001.docx]

**Table S1.** Definition of each contributing factor.

| **Contributing factor** | **Definition** |
| --- | --- |
| **Disease progression** | Disease progression in a patient who was clinically stable at discharge |
| **New medical problem** | New medical problem which was not anticipated |
| **Calculated risk** | The potential benefit of a treatment is estimated, by the health care professional, to outweigh the chance or severity of a known harmful (side) effect |
| **Palliative care** | making appropriate treatment/management decisions, taking into consideration the particular end of life situation of a patient, while maximising comfort and no longer focusing on cure |
| **Patient related** | insufficient self-care, substance abuse, refusal of care or non-adherence |
| **Social support** | insufficient or no care from social support system |
| **Extramural factors** | readmission was caused by interventions, or lack thereof, by care providers outside the hospital |
| **Other** | if not categorised in one of the above |
