# Supplementary material for: Preventability of unplanned readmissions within 30 days of discharge. A cross-sectional, single-center study
Source: PLoS One. 2020 Apr 2;15(4):e0229940. doi: 10.1371/journal.pone.0229940 (PMC7117704; doi:10.1371/journal.pone.0229940)
Supplement: S2 Table — The table represents the number (%) of contributing factors for three groups: total population, non-preventable readmissions and possible preventable readmissions. (DOCX) [file pone.0229940.s002.docx]

**Table S2.** Factors contributing to PPRs^a^ vs non-PPRs^b^. The table represents the number (%) of contributing factors for three groups: total population, non-preventable readmissions and possible preventable readmissions.

| **CONTRIBUTING FACTORS^a^** | | | |
| --- | --- | --- | --- |
|  | **Total population (n=430)** | **Non-preventable readmissions (n=374)** | **Possible preventable readmissions (n=56)** |
| **Disease progression** | 176 (31.3) | 161 (32.7) | 15 (21.7) |
| **New medical problem** | 55 (9.8) | 51 (10.3) | 4 (5.8) |
| **Calculated risk** | 170 (30.2) | 153 (31.0) | 17 (24.6) |
| **Palliative care** | 6 (1.1) | 4 (0.8) | 2 (2.9) |
| **Patient related** | 79 (14.1) | 63 (12.8) | 16 (23.2) |
| **Social support** | 41 (7.3) | 32 (6.5) | 9 (13) |
| **Extramural factors** | 21 (3.7) | 18 (3.7) | 3 (4.3) |
| **Other** | 14 (2.5) | 11 (2.2) | 3 (4.3) |
| **TOTAL** | 562 (100) | 493 (100) | 69 (100) |

^a^There could be ≥ 1 contributing factor per readmission

Supplementary Table S2 shows the contributing factors divided into 3 groups: low causation (score < 4), high causation (score ≥ 4) but low preventability (score < 4) and potentially preventable readmission (PPR) (score ≥ 4). The most reported contributing factors were disease progression, calculated risk, patient related factors and a new medical problem. The contribution per factor varied slightly between the groups.
