# Supplementary material for: Preventability of unplanned readmissions within 30 days of discharge. A cross-sectional, single-center study
Source: PLoS One. 2020 Apr 2;15(4):e0229940. doi: 10.1371/journal.pone.0229940 (PMC7117704; doi:10.1371/journal.pone.0229940)
Supplement: S3 Table — Reviewers used a six-point ordinal scale to rate whether the readmission was causal (readmission due to medical care during the index admission) and whether the readmission could have been prevented. (DOCX) [file pone.0229940.s003.docx]

Reviewers used a six-point ordinal scale to rate whether the readmission was causal (readmission due to medical care during the index admission) and whether the readmission could have been prevented.

**Table S3.** Causation and preventability scoring tools [12].

| **RATING** | **CAUSATION (≥ 4 is positive for causality)**  Question: Was the readmission caused by received care, a healthcare professional or healthcare organisation? | **PREVENTABILITY (score ≥ 4 is a potential preventable readmission)**  Question: In hindsight, how likely was it that this readmission could have been prevented by hospital care? |
| --- | --- | --- |
| 1 | No evidence for causation | No evidence for preventability |
| 2 | Slight evidence for causation | Slight evidence for preventability |
| 3 | Causation less than  50–50 but close call | Preventability less than  50–50 but close call |
| 4 | Causation more than  50–50 but close call | Preventability more than  50–50 but close call |
| 5 | Strong evidence for causation | Strong evidence for preventability |
| 6 | Near certain evidence for causation | Near certain evidence for preventability |
