# Supplementary material for: Preventability of unplanned readmissions within 30 days of discharge. A cross-sectional, single-center study
Source: PLoS One. 2020 Apr 2;15(4):e0229940. doi: 10.1371/journal.pone.0229940 (PMC7117704; doi:10.1371/journal.pone.0229940)
Supplement: S4 Table — Cause categories for readmissions and examples for each category. (DOCX) [file pone.0229940.s004.docx]

**S5 table.** Cause classification. Cause categories for readmissions and examples for each category.

| **Cause category** | **Example** |
| --- | --- |
| Surgical complication | Wound infection, post-surgical bleeding |
| Non-surgical procedural complication | Complications after diagnostic biopsy or puncture or pacemaker problems |
| Nosocomial infection (non-surgical) | Clostridium difficile |
| Medication error | Adverse drug event, more medications than the patient can manage |
| Diagnostic error | Incorrect or delayed diagnosis |
| Management | Clinical errors (i.e., lack of coordination of care), follow-up (i.e., no or delayed follow-up visit), premature discharge, no or lack of communication between care providers (in or outside the hospital), insufficient patient education. |
| System error | Insufficient facilities, lack of guidelines and/or insufficient workflow. |
| Other | Not elsewhere classified. |
