# Supplementary material for: Preventability of unplanned readmissions within 30 days of discharge. A cross-sectional, single-center study
Source: PLoS One. 2020 Apr 2;15(4):e0229940. doi: 10.1371/journal.pone.0229940 (PMC7117704; doi:10.1371/journal.pone.0229940)
Supplement: S1 File — (DOCX) [file pone.0229940.s005.docx]

**Supplementary information S4**. Sources used by residents for the causation and preventability assessment.

The main sources mentioned by resident reviewers for the causation and preventability assessment of the 430 readmissions were: the hospital admission report (21%), the discharge report (14%), notes made by physicians during hospital admission (10%), pre-assessment knowledge about patient and provided hospital care (7%) and other sources (32%), such as notes made by nurses, laboratory and vital signs, outpatient notes, patient or family reported information in daily practice and information gained from consulted experts. In 16% of readmissions, the source was not specified.
