# Supplementary material for: Preventability of unplanned readmissions within 30 days of discharge. A cross-sectional, single-center study
Source: PLoS One. 2020 Apr 2;15(4):e0229940. doi: 10.1371/journal.pone.0229940 (PMC7117704; doi:10.1371/journal.pone.0229940)
Supplement: S2 File — (DOCX) [file pone.0229940.s006.docx]

**Vragenlijst ongeplande heropnames**

1. U bent van ________ tot ________ opgenomen geweest. Waarom was u toen opgenomen?

__________________________________________________________________________________

___________________________________________________________________________________

1. Wat is volgens u de reden dat u opnieuw bent opgenomen?

__________________________________________________________________________________

___________________________________________________________________________________

***Interviewer****: Ik wil nu teruggaan naar uw vorige ziekenhuisopname om inzicht te krijgen in welke zorgverleners (i.e. artsen, verpleegkundigen) betrokken zijn geweest na uw ontslag.*

1. Kreeg u bij uw laatste ontslag een telefonische afspraak of vervolgafspraak op de polikliniek van OLVG?

⬜ Ja, telefonisch: ga naar vraag 5a

⬜ Ja, fysiek: ga naar vraag 5b

⬜ Nee: ga naar vraag 5c

- 1. Was een telefonische afspraak voldoende of had u liever een afspraak in het ziekenhuis gehad?

⬜ Ja, was voldoende ⬜ Nee, omdat ________________________________________

- 1. Bent u al op deze vervolgafspraak geweest?

⬜ Ja ⬜ Nee, omdat ________________________________________

- 1. Had u het gevoel dat een vervolgafspraak of telefonisch overleg wel nodig was?

⬜ Nvt ⬜ Ja, een vervolgafspraak/ telefonisch overleg (omcirkel wat van toepassing is) omdat ___________________________________________________________

⬜ Nee, omdat_________________________________________________________________

1. Heeft u na uw laatste ontslag uit dit ziekenhuis nog (telefonisch/fysiek) contact gehad met een zorgverlener **die niet werkt in** OLVG, locatie West?

|  | 1. Telefonisch | 2. Fysiek |
| --- | --- | --- |
| A. ⬜ Nee |  |  |
| B. ⬜ Ja, met mijn **huisarts**  C. ⬜ Ja, met de dienstdoende **huisarts/huisartsenpost** |  |  |
| D. ⬜ Ja, de **SEH** van het ziekenhuis_________________________ |  |  |
| E. ⬜ Ja, een **opname** in het ziekenhuis _____________________ |  |  |
| F. ⬜ Ja, een **poliklinisch consult** bij het specialisme ______________ in het ziekenhuis _________________________ |  |  |
| G. ⬜ Ja, anders ________________________________________ |  |  |

____________________________________________________________________________________

**INTERVIEWER: GA NAAR DE GEZONDHEIDSVAARDIGHEDEN en B-PREPARED VRAGENLIJSTEN pagina 5, vragen 7 t/m 20 en vervolg daarna met onderstaande vragen.**

1. Vond u dat u te vroeg naar huis mocht na bij de vorige opname?

⬜ Nee ⬜ Ja, namelijk _____________________________________________________

1. Kreeg u bij uw vorige ziekenhuisopname dieet- en/of leefstijladviezen? (Bijvoorbeeld een vochtbeperking of een beperkte zoutinname of het advies om te bewegen)

⬜ Nee ⬜ Ja, namelijk ________________________________ ⬜ Weet ik niet meer

- 1. Indien van toepassing, lukte het u om aan deze adviezen te houden?

⬜ Ja ⬜ Nee, omdat _____________________________________________________

1. Hebt u voordat u het ziekenhuis verliet schriftelijke en/of mondelinge informatie gekregen van artsen, verpleegkundigen of ander ziekenhuispersoneel, over wat u moet doen als er thuis problemen optreden?

⬜ Nee ⬜ Ja ⬜ Weet ik niet meer

***Interviewer:*** *Ik wil het nu met u hebben over de medicijnen die u nu thuis gebruikt.*

1. Hoeveel verschillende medicijnen gebruikt u thuis? _______________________________________________________________________________
2. Gebruikt u ook medicijnen die u zonder recept koopt bij een drogist, apotheek of natuurwinkel? *(bijvoorbeeld vitamines,* *middelen tegen de pijn , rustgevende middelen, kruiden, homeopathische middelen, online gekocht)*

⬜ Nee ⬜ Ja, namelijk ______________________________________________________

1. Krijgt u thuis hulp bij uw medicijngebruik?

⬜ Ja, thuiszorg ⬜ Ja, familielid of _____________­­­­­­___ ⬜ Ja, weekdoos/baxterrol ⬜ Nee

1. Bij uw vorige opname in OLVG West: zijn er toen medicijnen aangepast?

| a. Is er (een) nieuwe medicijn(en) gestart? *(die u voor de opname nog niet gebruikte)* | ⬜ nee | ⬜ weet  niet | ⬜ ja, nl (naam/namen medicijn) _______________________________ |
| --- | --- | --- | --- |
| b. Is er een dosis of schema aangepast? *(hoeveelheid/sterkte van het medicijn*) | ⬜ nee | ⬜ weet  niet | ⬜ ja, nl(naam/namen medicijn) _______________________________ |
| c. Is er een medicijn vervangen door een ander medicijn | ⬜ nee | ⬜ weet  niet | ⬜ ja, nl (naam/namen medicijn) _______________________________ |
| d. Is er een medicijn die u voor de opname gebruikte gestopt? | ⬜ nee | ⬜ weet  niet | ⬜ ja, nl (naam/namen medicijn) ______________________________ |

1. Lukte het u om de gewijzigde medicatie thuis te gebruiken volgens het voorschrift?

⬜ Nvt ⬜ Ja ⬜ Nee, __________________________________________

1. Is er **na** uw ziekenhuisopname van _______ tot _____ nog iets gewijzigd aan uw medicijnen? *(gestart, dosis aangepast, ander medicijn, gestopt)*

⬜ Nvt ⬜ Ja, namelijk ______________________________________door ___________

1. Denkt u dat uw medicijnen eraan hebben bijgedragen dat u weer naar het ziekenhuis moest? (bijv. bijwerking, teveel geneesmiddelen)

⬜ Nee ⬜ Ja, namelijk _____________________________________________________

***Interviewer****: Mensen die veel geneesmiddelen gebruiken, slaan wel eens een keer over. Hiervoor kunnen verschillende redenen zijn. Bent u het eens bent met de volgende uitspraken?*

| 1. *Ik sla wel eens een keer het nemen van mijn* | helemaal | niet | geen | mee | helemaal |
| --- | --- | --- | --- | --- | --- |
| ***medicijn(en)*** *over omdat:* | niet | mee | duidelijke | eens | mee |
|  | mee eens | eens | mening |  | eens |
| a. Ik niet weet waarom ik ze nu eigenlijk moet gebruiken | ⬜ | ⬜ | ⬜ | ⬜ | ⬜ |
| b. Ik het te druk heb om eraan te denken | ⬜ | ⬜ | ⬜ | ⬜ | ⬜ |
| c. Ik geen last wil hebben van bijwerkingen | ⬜ | ⬜ | ⬜ | ⬜ | ⬜ |
| d. Ik geen regelmatig dagritme heb | ⬜ | ⬜ | ⬜ | ⬜ | ⬜ |
| e. Ik ze niet nuttig vind | ⬜ | ⬜ | ⬜ | ⬜ | ⬜ |
| f.  Iemand anders me er niet aan herinnert | ⬜ | ⬜ | ⬜ | ⬜ | ⬜ |
| g. Het gebeurt zonder een duidelijke reden | ⬜ | ⬜ | ⬜ | ⬜ | ⬜ |
| h. Andere reden, nl: ………………………………. | ⬜ | ⬜ | ⬜ | ⬜ | ⬜ |

***Interviewer:*** *Ik wil nu dieper ingaan op hoe u de periode na de ziekenhuisopname van ______tot_______ ervaren hebt.*

1. Welke problemen en/of onduidelijkheden heeft u ervaren **na** uw ziekenhuisopname? (*bijv. onvoldoende nazorg, problemen in dagelijkse activiteiten zoals baden, eten, naar toilet gaan*)

⬜ N.v.t. ⬜ De problemen, __________________________________________________

Indien ja, was een heropname mede vanwege die problemen noodzakelijk?

⬜ Nee ⬜ weet niet ⬜ ja___________________________________________

1. Was er iemand beschikbaar die voor u kon zorgen na uw ziekenhuisopname zodat u kon herstellen? (hulp bij het huishouden, hulp bij persoonlijke verzorging, verpleging)

⬜ Ja ⬜ Dat was niet nodig ⬜ Nee, ________________________________________

1. Vindt u het wel eens lastig om zelf om hulp te vragen?

⬜ Ja ⬜ Nee

1. Zou u willen dat er meer mensen waren waar u mee kon praten en die u emotionele steun zouden kunnen geven na uw ziekenhuisopname?

⬜ Nvt ⬜ Dat was niet nodig ⬜ Ja, __________________________________________

1. Had u verwacht dat u weer opgenomen zou worden in het ziekenhuis?

⬜ Nee ⬜ Ja, namelijk _____________________________________________________

1. Als u terugkijkt naar uw laatste opname, is er dan iets wat uw huisarts, het ziekenhuis, uw familie of uzelf anders hadden kunnen doen zodat u niet weer opgenomen zou zijn in het ziekenhuis?

⬜ Nee ⬜ Ja, namelijk

⬜ Huisarts ______________________________________________

⬜ Ziekenhuis ______________________________________________

⬜ Familie ______________________________________________

⬜ Zelf ______________________________________________

⬜ Anders ______________________________________________

***Interviewer:*** *Ik wil u nu enkele algemene vragen stellen.*

1. Wat is uw woonsituatie op dit moment?

⬜ Ik woon alleen ⬜ Ik woon samen

1. In welk land bent u geboren?

⬜ Nederland ⬜ Suriname ⬜ NL Antillen ⬜ Turkije ⬜ Marokko ⬜ Anders, nl: ____________

1. In welk land is uw moeder geboren?

⬜ Nederland ⬜ Suriname ⬜ NL Antillen ⬜ Turkije ⬜ Marokko ⬜ Anders, nl: ____________

1. In welk land is uw vader geboren?

⬜ Nederland ⬜ Suriname ⬜ NL Antillen ⬜ Turkije ⬜ Marokko ⬜ Anders, nl: ____________

1. Wat is uw hoogst voltooide opleiding?

⬜ Geen opleiding afgerond

⬜ Lagere school/basisonderwijs

⬜ Lager beroepsonderwijs (zoals huishoudschool, LTS, LEAO)

⬜ Middelbaar algemeen voortgezet onderwijs (zoals MAVO, (M)ULO)

⬜ Middelbaar beroepsonderwijs (zoals MBO, MTS, MEAO, MHNO, INAS)

⬜ Hoger algemeen voortgezet onderwijs (zoals HAVO, VWO, HBS, MMS)

⬜ Hoger beroepsonderwijs (zoals HBO, HTS, HEAO, PABO)

⬜ Wetenschappelijk onderwijs (universiteit)

⬜ Anders, namelijk……………………………………………

1. Wat vindt u, over het algemeen, van uw gezondheid?
2. Uitstekend
3. Zeer goed
4. Goed
5. Matig
6. Slecht

**Deel voor Interviewer:**

De vragenlijst is afgenomen met:

⬜ Patiënt zelf ⬜ Patiënt/mantelzorger ⬜ Mantelzorger

Heb je een taalbarrière ervaren met de patiënt/mantelzorger:

⬜ Nee ⬜ Ja, namelijk _____________________________________________________

Hoe lang duurde het interview in minuten: _________________________________________________

Opmerkingen/ bijzonderheden/ notities

⬜ Nee ⬜ Ja, namelijk _____________________________________________________

_____________________________________________________________________________________

_____________________________________________________________________________________

***GEZONDHEIDSVAARDIGHEDEN VRAGENLIJST***

***Interviewer:*** *Ik wil u nu drie vragen stellen over uw ervaring met (medische) formulieren.*

1. Hoe vaak helpt iemand u met het lezen van brieven of folders van uw huisarts, het ziekenhuis of andere zorginstellingen?
2. Nooit c. Soms e. Altijd
3. Af en toe d. Vaak
4. Hoe zeker bent u ervan dat u medische formulieren zelf goed invult?
5. Heel erg c. Een beetje e. Helemaal niet
6. Nogal d. Een klein beetje
7. Vindt u het moeilijk om meer te weten te komen over uw gezondheid, omdat u geschreven informatie niet goed begrijpt?
8. Nooit c. Soms e. Altijd
9. Af en toe d. Vaak

***B-PREPARED VRAGENLIJST***

***Interviewer****: Ik wil uw ervaringen over uw vorige ziekenhuisopname van ______ tot _______bespreken.*

1. Hoeveel informatie heeft u toen u in het ziekenhuis lag ontvangen over de medicijnen die u thuis moest innemen?
2. Geen informatie
3. Enige informatie, maar niet genoeg
4. Zoveel informatie als ik nodig had of ik neem geen medicijnen in
5. Hoeveel informatie heeft u toen u in het ziekenhuis lag ontvangen over de bijwerkingen van de medicijnen die u thuis moest innemen?
6. Geen informatie
7. Enige informatie, maar niet genoeg
8. Zoveel informatie als ik nodig had of ik neem geen medicijnen in.
9. Heeft u schriftelijke instructies gekregen met betrekking tot uw medicijnen toen u in het ziekenhuis lag? Zo ja, heeft er iemand de tijd genomen om de schriftelijke instructies uit te leggen?
10. Ik heb geen schriftelijke instructies ontvangen en niemand heeft daarvoor de tijd genomen
11. Ja, ik heb schriftelijke instructies ontvangen maar niemand heeft daarvoor de tijd genomen
12. Ja, ik heb schriftelijke instructies ontvangen en ja, iemand heeft daarvoor de tijd genomen of ik neem geen medicatie in
13. Hoeveel informatie heeft u toen u in het ziekenhuis lag ontvangen over hoe u uw dagelijkse activiteiten kunt voortzetten wanneer u weer thuis bent?
14. Geen informatie
15. Enige informatie, maar niet genoeg
16. Zoveel informatie als ik nodig had.
17. Hoeveel informatie heeft u toen u in het ziekenhuis lag ontvangen over de zorg voor in uw thuissituatie (zoals thuiszorg) waar u gebruik van kunt maken?
18. Geen informatie
19. Enige informatie, maar niet genoeg
20. Zoveel informatie als ik nodig had of ik heb geen zorg thuis nodig.
21. Hoeveel informatie heeft u toen u in het ziekenhuis lag ontvangen over hulpmiddelen (oa apparatuur) die u nodig zou kunnen hebben wanneer u weer thuis bent?
22. Geen informatie
23. Enige informatie, maar niet genoeg
24. Zoveel informatie als ik nodig had of ik heb geen hulpmiddelen nodig.
25. Heeft er iemand de zorg voor in uw thuissituatie (zoals thuiszorg) voor u geregeld toen u werd ontslagen uit het ziekenhuis?
26. Nee
27. Ja
28. Niemand heeft dit hoeven regelen omdat het al geregeld was of ik heb geen voorzieningen nodig.
29. Heeft er iemand hulpmiddelen (oa apparatuur) voor in uw thuissituatie voor u geregeld voordat u werd ontslagen uit het ziekenhuis?
30. Nee
31. Ja
32. Niemand heeft dit hoeven regelen omdat ik al hulpmiddelen had of ik heb geen hulpmiddelen nodig.
33. Had u voordat u uit het ziekenhuis werd ontslagen andere informatie willen krijgen om u voor te bereiden om thuis zelfstandig te kunnen functioneren?
34. Nee
35. Ja, nl ____________________________________________________________________
36. Hoeveel vertrouwen had u er in dat u het thuis zou redden nadat u het bericht kreeg dat u het ziekenhuis mocht verlaten?
37. Geen vertrouwen
38. Onzeker
39. Vol vertrouwen
40. Als u terugkijkt naar het moment waarop u het ziekenhuis verliet, hoe voorbereid voelde u zich toen in het algemeen om terug naar huis te gaan?
41. Onvoorbereid
42. Redelijk goed voorbereid
43. Goed voorbereid
