# Supplementary material for: Preventability of unplanned readmissions within 30 days of discharge. A cross-sectional, single-center study
Source: PLoS One. 2020 Apr 2;15(4):e0229940. doi: 10.1371/journal.pone.0229940 (PMC7117704; doi:10.1371/journal.pone.0229940)
Supplement: S3 File — (DOCX) [file pone.0229940.s007.docx]

**Questions for the patient:**

1. You were admitted to the hospital from______ to ______. Why were you admitted to the hospital back then? ________________________________________________________________________________
2. What do you think that the reason is that you are readmitted?

________________________________________________________________________________

***Interviewer****: I would like to go back to your previous admission to gain insight into which healthcare professionals (i.e. physicians, nursing staff) were involved after your discharge from the hospital.*

1. At the time of your previous discharge, were you given a follow up appointment either as a telephonic consultation or at one of the outpatient departments of OLVG?

⬜ Yes, telephonically, go to question 5a

⬜ Yes, physically at one of the outpatient departments. Go to question 5b

⬜ No; go to question 5C.

- 1. Was a telephonic appointment sufficient or would you have preferred to have had an appointment at the hospital?

⬜ Yes, it was sufficient ⬜ No, because__________________________________

- 1. Have you attended this appointment already?

⬜ Yes ⬜ No, because __________________________________

- 1. Did you feel a follow up appointment or telephone call was necessary?

⬜ not applicable ⬜ Yes, a follow up appointment was necessary because: ___________________________________________________________

⬜ No, because_________________________________________________________________

1. After your last discharge from this hospital, have you been in contact with any other health care professional, **not working at OLVG**, location West?

|  | 1. By phone | 2. In person |
| --- | --- | --- |
| A. ⬜ No |  |  |
| B. ⬜ Yes, with my **GP**  C. ⬜ Yes, with the **attending GP** after hours |  |  |
| D. ⬜ Yes, with the **emergency department** of ______________ |  |  |
| E. ⬜ Yes, an **admission** to hospital _____________________ |  |  |
| F. ⬜ Yes, an **outpatient consultation** with the department of ______________ at _________________________hospital. |  |  |
| G. ⬜ Yes, but none of the above, but:_______________________ |  |  |

____________________________________________________________________________________

**INTERVIEWER: GO TO THE HEALTH LITERACY QUESTIONNAIRE AND B-PREPARED QUESTIONNARE ON PAGE 5-7 (questions 7-20) and then continue with the questions below.**

1. Do you feel you were discharged prematurely during the precious admission?

⬜ No ⬜ Yes, because _____________________________________________________

1. Were you given dietary or life style advise during your previous admission? For example; a fluid restriction or limited salt intake or an advice regarding physical activity.

⬜ No ⬜ Yes, namely ________________________________ ⬜ I cannot remember

- 1. If applicable, were you able to adhere to this advice?

⬜ Yes ⬜ No, because _____________________________________________________

1. Prior to leaving the hospital, have you received any oral or written information from a healthcare worker regarding what to do in case of problems occurring at home?

⬜ No ⬜ Yes ⬜ I cannot remember

***Interviewer:*** *Now, I would like to discuss the medication you use at home.*

1. How many different medications do you use at home? _______________________________________________________________________________
2. Do you use any over the counter medication? *(For example, vitamins, analgesics, calming agents, herbal medication, homeopathic remedies or medication from online webshops)*.

⬜ No ⬜ Yes, namely ______________________________________________________

1. At home, do you receive help with taking medication?

⬜ Yes, by home based care services ⬜ Yes, relative or _____________­­­­­­___ ⬜ Yes, I receive my medication per week box or in a multi dose drug dispensing system ⬜ No

1. Have there been adjustments regarding your medication during your last admission to OLVG?

| a. Was a new medicine started, not used prior to admission? | ⬜ No | ⬜ I do not know | ⬜ yes, the following (name of medication) _______________________________ |
| --- | --- | --- | --- |
| b. Has there been an adjustment to the dose of any medication? *(amount/dosage of the medication)* | ⬜ No | ⬜ I do not know | ⬜ yes, the following (name of medication) _______________________________ |
| c. Has any medication been replaced by another? | ⬜ No | ⬜ I do not know | ⬜ yes, the following (name of medication) _______________________________ |
| d. Has any of the medication that you used prior to admission been stopped? | ⬜ No | ⬜ I do not know | ⬜ yes, the following (name of medication) ______________________________ |

1. Did you manage to use the altered medications according to the new prescription at home?

⬜ Not applicable ⬜ Yes ⬜ No, _____________________________

1. Were there any changes made to your medication **after** your hospitalization from ______until _____? *(start, dose adjustments, different medicine, stop)*

⬜ Not applicable ⬜ Yes, the following: __________________________________

1. Do you feel that your medication contributed to you having to go to the hospital again? For example: side effects, too many medicines?

⬜ No ⬜ Yes, the following: __________________________________

***Interviewer****: People that have to use many medicines, might skip a dose. There can be many reasons for this. Do you agree with the following statements?*

| 1. *I sometimes skip taking my* ***medications*** *at times because:* | completely disagree | disagree | neutral | agree | completely agree |
| --- | --- | --- | --- | --- | --- |
|  |  |  |  |  |  |
|  |  |  |  |  |  |
| a. I do not know what they are for | ⬜ | ⬜ | ⬜ | ⬜ | ⬜ |
| b. II am too busy to think of it | ⬜ | ⬜ | ⬜ | ⬜ | ⬜ |
| c. II do not want to suffer from side effects | ⬜ | ⬜ | ⬜ | ⬜ | ⬜ |
| d. I do not have a regular daily routine | ⬜ | ⬜ | ⬜ | ⬜ | ⬜ |
| e. I do not feel they are useful | ⬜ | ⬜ | ⬜ | ⬜ | ⬜ |
| f.  Someone else does not remind me | ⬜ | ⬜ | ⬜ | ⬜ | ⬜ |
| g. It happens without any clear reason | ⬜ | ⬜ | ⬜ | ⬜ | ⬜ |
| h. Different reason: ………………………………. | ⬜ | ⬜ | ⬜ | ⬜ | ⬜ |

***Interviewer:*** *I would like to discuss how you experienced the period after your admission from ______to _______.*

1. What problems/ issues have you experienced after your hospital admission? For example: insufficient after care, problems with regards to activities of daily living like bathing, eating, going to the toilet.

⬜ not applicable. ⬜ yes, the following, _______________________________________

If yes, was a readmission needed due to those problems?

⬜ No ⬜ I do not know ⬜ yes_____________________________________

1. Was there anyone available who could care for you in order for you to recover? *(Help with housekeeping, or personal care, nursing care)*?

⬜ Yes ⬜ That was not necessary ⬜ No, _____________________________________

1. Do you find it difficult, sometimes, to ask for help?

⬜ Yes ⬜ No

1. Would you want that more people were available to talk to and that could give emotional support after your admission?

⬜ Not applicable ⬜ that was not necessary ⬜ Yes, _____________________________________

1. Had you expected to be readmitted to the hospital?

⬜ No ⬜ Yes, because _____________________________________________________

1. If you look back at your last admission, is there something that your doctor, the hospital, your family or yourself could have done differently so that you would not have been admitted to the hospital again?

No Yes, namely

General practitioner _____________________________________________

Hospital _____________________________________________

Family _____________________________________________

Self _____________________________________________

Other _____________________________________________

***Interviewer****: I would like to ask you some general questions.*

1. What is your living situation at the moment?

I live alone I live together

1. In which country were you born?

The Netherlands Suriname NL Antilles Turkey Morocco Other, namely: ____________

1. In which country was your mother born?

The Netherlands Suriname NL Antilles Turkey Morocco Other, namely: ____________

1. In which country was your father born?

The Netherlands Suriname NL Antilles Turkey Morocco Other, namely: ____________

1. What is your highest completed education?

No training completed

Primary school

Secondary general education (such as MAVO, (M) ULO)

(Lower) vocational education (such as LTS, MBO, MTS, MEAO, MHNO, INAS)

Higher general secondary education (such as HAVO, VWO, HBS, MMS)

Higher professional education (such as HBO, HTS, HEAO, PABO)

University

Other, namely ……………………………………………

1. In general, would you say your health is:

a. Excellent

b. Very good

c. Good

d. Fair

e. Poor

**To be completed by the Interviewer:**

The questionnaire was completed with:

Patient himself Patient / caregiver Caregiver

Have you experienced a language barrier with the patient / caregiver?

No Yes, namely __________________________________________________________________

How long did the interview last in minutes? _________________________________________________

Remarks / details / notes

No Yes, namely ___________________________________________________________________

***HEALTH LITERACY QUESTIONNAIRE***

***Interviewer:*** *I would like to ask you about your experience with (medical) forms;*

1. How often do you receive help with reading letters/flyers from your GP, the hospital or other healthcare institutes?
   1. never c. sometimes e. always
   2. every now and then d. often
2. How sure are you of filling out medical forms correctly yourself?
   1. Very sure c. a little e. not at all
   2. Reasonably sure d. slightly
3. Do you find it hard to find out more about your health due to not understanding written information properly?
4. Never c. Sometimes e. Always
5. Every now and then d. Often

***B-prepared questionnaire was used completely. See:***

1. Graumlich JF, Novotny NL, Aldag JC. Brief scale measuring patient preparedness for hospital discharge to home: Psychometric properties. J Hosp Med. 2008 Nov-Dec;3(6):446–54. doi: 10.1002/jhm.316.
