# Supplementary material for: Preventability of unplanned readmissions within 30 days of discharge. A cross-sectional, single-center study
Source: PLoS One. 2020 Apr 2;15(4):e0229940. doi: 10.1371/journal.pone.0229940 (PMC7117704; doi:10.1371/journal.pone.0229940)
Supplement: S2 Fig — (DOCX) [file pone.0229940.s010.docx]

S9: causes of PPR by department (n=57)
