# Supplementary material for: Preventability of unplanned readmissions within 30 days of discharge. A cross-sectional, single-center study
Source: PLoS One. 2020 Apr 2;15(4):e0229940. doi: 10.1371/journal.pone.0229940 (PMC7117704; doi:10.1371/journal.pone.0229940)
Supplement: Supplementary file 9 [file pone.0229940.s009.docx]

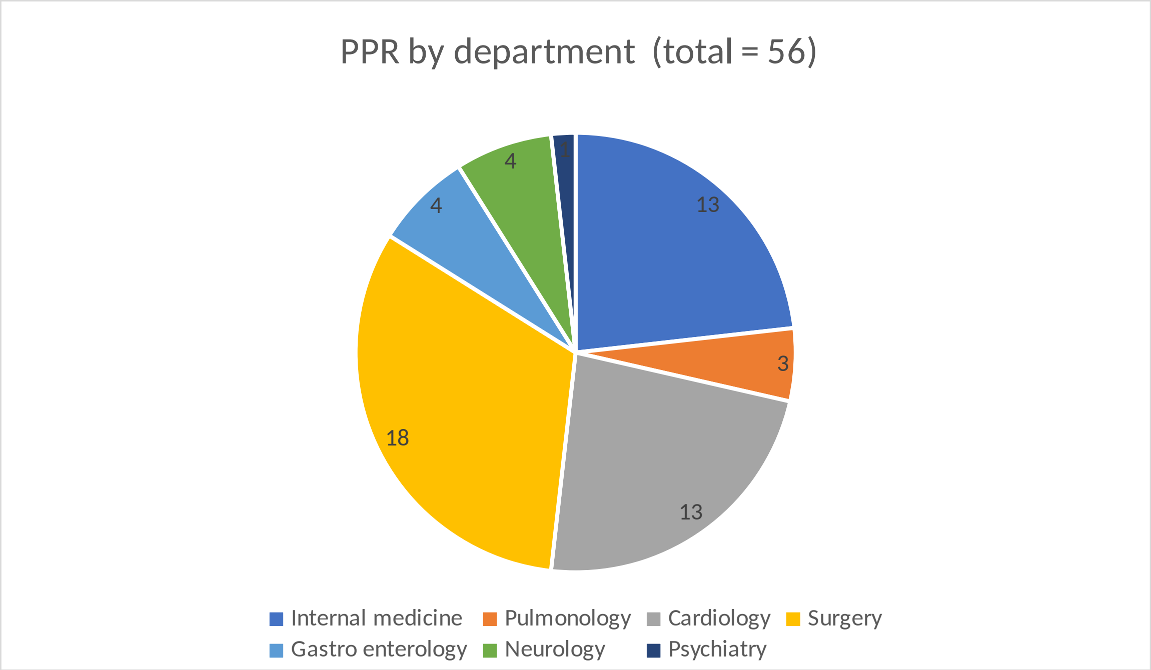
PPR by department (total = 56)
13
3
13
18
4
4
Internal medicine
Pulmonology
Cardiology
Surgery
Gastro enterology
Neurology
Psychiatry
