# Supplementary material for: Preventability of unplanned readmissions within 30 days of discharge. A cross-sectional, single-center study
Source: PLoS One. 2020 Apr 2;15(4):e0229940. doi: 10.1371/journal.pone.0229940 (PMC7117704; doi:10.1371/journal.pone.0229940)
Supplement: Attachment — Submitted filename: Response to reviewers .docx [file pone.0229940.s011.docx]

**Response to Editor’s and Reviewer’s comments**

Preventability of unplanned readmissions within 30 days of discharge: A cross-sectional, single-centre study.PONE-D-19-31179

We would like to thank the reviewers for their comments, which helped us to improve the manuscript. We respond on each comment and explain which adjustments were made.

Kind regards, on behalf of all co-authors,

Albertine van der Does

Amsterdam, 14^th^ of February

| **Editor’s and Reviewer’s Comments** | **Our Response** | **Location of edits** |
| --- | --- | --- |
| **Journal requirements** |  |  |
| 1. Please ensure that your manuscript meets PLOS ONE's style requirements, including those for file naming. | The manuscript was checked according to the style requirements, but no discrepancies were found. |  |
| 2. Interview guide; For instance, if you developed a guide as part of this study and it is not under a copyright more restrictive than CC-BY, please include a copy, in both the original language and English, as Supporting Information." | Two supporting information files were created; the Dutch interview guide (S6 file) and the English translation (S7 file) of that file. | Supporting information: S6 file and S7 file |
| 3 a) Please amend your current ethics statement to include the full name of the ethics committee/institutional review board(s) that approved your specific study. | The study was approved by the Advisory Committee Scientific Research of OLVG hospital (Advies Commissie Wetenschappelijk Onderzoek). Informed consent was obtained from patients for the interview and to contact the community pharmacist or general practitioner for additional information (e.g., on medication adherence). | Line number 103-104 in Methods section |
| 3 b) Once you have amended this statement in the Methods section of the manuscript, please add the same text to the “Ethics Statement” field of the submission form (via “Edit Submission”). | done |  |
| 4. If you wish to make changes to your Data Availability statement, please describe these changes in your cover letter and we will update your Data Availability statement to reflect the information you provide. | We do not wish to make changes to our Data Availability statement. |  |
| **Reviewer 1** |  |  |
| My one concern about the study design was with the Causation and preventability tool, (Table S3). This was the tool used to determine inclusion into the study and was based on subjective assessments of prior hospital care and was scored by the discharging team. Thus, this leaves the possibility of underscoring patients who might otherwise meet criteria and thus artificially lowering the percentage of PPR. This should be addressed in the discussion. | Thank you for this comment. This is indeed correct. We have added this to the strength and weaknesses paragraph: *Inclusion was determined by subjective assessment by a member of the discharging department. This could lead to non-inclusion of patients who might have had a PPR, thus preventable readmission cases could therefore have been underestimated.* | Line number 333-333-335 |
| **Reviewer 2** |  |  |
| but I would prefer that this project is either stratified based on specialty or you could split the project into 2 or 3 papers based on the different specialties. Reasons of readmission would be different in surgery from medicine. So, putting both in one basket, would affect your results. I would rather put cardiology, gastroenterology, internal medicine, neurology, pulmonology in one group and general surgery in a separate group. Psychiatry also would have different group by itself. | We agree that patients from different specialties are not fully comparable. However, the number of (possible preventable) readmissions per specialty was limited (see S8), making it difficult to draw firm conclusions. We wanted to give a broad overview of the preventability of readmissions, but to give insight per specialty we have created S9 illustrating causes of PPR per department. | Supporting information: S8 and S9 |
